# Cytotoxic and proapoptotic effects of alizarin in mice with Ehrlich solid tumor: novel insights into ERα-mediated MDM2/p-Rb/E2F1 signaling pathway

**DOI:** 10.1007/s00210-025-04196-7

**Published:** 2025-06-28

**Authors:** Aya H. Eid, Ahmed A. Al-Karmalawy, Reda F. A. Abdelhameed, Yasser M. Mostafa, Gouda K. Helal, Eman T. Mehanna, Reem M. Hazem

**Affiliations:** 1 Department of Pharmacology & Toxicology, Faculty of Pharmacy, Heliopolis University, Cairo, 11785 Egypt; 2 Department of Pharmaceutical Chemistry, Collage of Pharmacy, The University of Mashreq, Baghdad, 10023 Iraq; 3 Department of Pharmaceutical Chemistry, Faculty of Pharmacy, Horus University-Egypt, New Damietta, 34518 Egypt; 4 Department of Pharmacognosy, Faculty of Pharmacy, Suez Canal University, Ismailia, 41522 Egypt; 5 Department of Pharmacognosy, Faculty of Pharmacy, Galala University, New Galala, 43713 Egypt; 6 Department of Pharmacology & Toxicology, Faculty of Pharmacy, Suez Canal University, Ismailia, 41522 Egypt; 7 Department of Pharmacology & Toxicology, Faculty of Pharmacy, Badr University in Cairo, Cairo, 11829 Egypt; 8 Department of Pharmacology & Toxicology, Faculty of Pharmacy, Al Azhar University, Cairo, 11651 Egypt; 9 Department of Biochemistry, Faculty of Pharmacy, Suez Canal University, Ismailia, 41522 Egypt

**Keywords:** Breast cancer, Alizarin, Ehrlich solid tumor, MDM2, ERα

## Abstract

The role of estrogen receptor (ER) and its related effects is crucial for growth of breast cancer cells. This study aimed to assess the cytotoxic impact of alizarin in models of breast cancer in vivo and investigate its antiestrogenic and apoptotic properties. MTT assay was used to evaluate alizarin cytotoxic effect against MCF7 and MDA-MB-231. In vivo, 30 mice were insulted with Ehrlich tumor cells. They were randomly allocated into 3 groups, 10 mice each. Alizarin was orally administered to two treatment groups (50 mg/kg and 100 mg/kg), respectively. The third group was considered as a positive control group. Western blot was used to evaluate the expression of mouse double minute 2 (MDM2), phosphorylated retinoblastoma (pRb), and E2F1. Caspase threefold change was measured by RT-PCR. ERα, Bax, and p53 expressions were investigated by immunohistochemistry. Molecular docking study of alizarin effect on ER was performed. Alizarin demonstrated dose dependent cytotoxicity against MCF7 and MDA-MB cell lines. Alizarin decreased tumor weight in mice, prompted cell cycle arrest, and stimulated cell apoptosis by impeding ERα-mediated tumorigenic effects, and inactivating its related MDM2/p-Rb/E2F1 signaling cascade with upregulation of target genes involved in cell apoptosis (Bax, caspase 3, and p53). Molecular docking proposed that alizarin is a very promising inhibitor to ER. Alizarin represented a promising approach to inhibit cell proliferation of breast cancer by modulating estrogen receptor mediated effects on MDM2/p-Rb/E2F1 axis concomitantly with activating apoptosis of cancer cells.

## Introduction

Breast cancer is the primary cause of cancer deaths globally and the second most common cancer in women after skin cancer (DeSantis et al. 2011). There are a number of core approaches for treating cancer comprising surgery, radiation therapy, immunologic therapy, and chemical-based therapies. Usually, a combination of these techniques is employed, and the majority of therapeutic strategies have a chemical component (Yildizhan et al. 2018). In most cases, drug resistance limits the effectiveness of the available treatment. Additionally, the numerous negative side effects of various synthetic medications make them a poor choice (Ghate et al. 2013). Consequently, phytocompounds have gained importance in the management of cancer, focusing on breast cancer especially (Ghate et al. 2013; Jameera Begam et al. 2017).

Most breast cancers show positive expression of estrogen receptor alpha (ERα +) which plays a significant role in tumor development (Martín 2006). It was reported that estrogen stimulates cell proliferation (Rodrik et al. 2005) of ERα + breast cancer cells like MCF7 (Brekman et al. 2011) and Ehrlich adenocarcinoma cells (Ozcan Arican and Ozalpan 2007). The human MDM2 gene is an oncogene that is overexpressed in various human malignances (Karni-Schmidt et al. 2016, Eid et al. 2023). The primary oncogenic role of MDM2 is to hinder the p53 tumor suppressor gene, resulting in abnormal cell growth and proliferation (Eid et al. 2023). The overexpression of MDM2 is detected in many ER + breast cancers, indicating that MDM2 is an essential ER + oncogene which may be targeted for cancer treatment (Brigham et al. 2012). In particular, the proliferative goals of MDM2 comprise the activation of E2 F1 transcriptional action (Kundu et al. 2017) amplified E2 F1 protein stability (Zhang et al. 2005), breakdown of the Rb-E2 F1 complex, and suppressing inhibitory effects of Rb on tumor (Miwa et al. 2006). Apoptosis inhibition is regarded as a crucial step in the progression of tumors. Therefore, most anticancer medications induce apoptosis by upregulating apoptotic genes including p53, Bax, and caspases 3, 7, and 9 and downregulating antiapoptotic genes such Bcl2 (Martin 2006).

The antitumor achievements of taxanes, vinca alkaloids, and their related derivatives have proved that natural products are a good source of novel anticancer agents (Vennila et al. 2012). Several anthraquinones have received much attention as potential sources of organic anticancer drugs. *Rubia* plant which is commonly known as madder from family Rubiaceae contains significant levels of anthraquinones; alizarin is one of these anthraquinones (Vankar et al. 2008). Due to anti-inflammatory, antioxidant, and antibacterial properties of alizarin, it was widely used in old Chinese medicine. Furthermore, alizarin and its derivatives have demonstrated important biological effects such as antileukemia, anti-HIV and antitumor activities (Luo et al. 2016).

Anticancer effect of alizarin has been studied against many cancer cells like SK-OV- 3, MGC- 803, Hep-G2, T24, NCI-H460 (Huang et al. 2017a, b), CEM-SS (Ali et al. 2000), MG- 63, U- 2 OS, and Soas- 2 (Fotia et al. 2012). Practically, alizarin has been used successfully to treat bone cancers (Ali et al. 2000; Fotia et al. 2012). However, the precise mechanism of action of alizarin has not been fully elucidated. Previous studies demonstrated that alizarin induces antiproliferative effects through the activation of Akt and extracellular signal-regulated kinase ERK pathways that cause S-phase cell cycle arrest, while decreasing the ratio of G0/G1 as well as G2/M phases. This cytostatic effect has been linked to its ability to inhibit tumorigenesis (Fotia et al. 2012), Additionally, in the 4 T1 triple-negative breast cancer (TNBC) cell line, alizarin has been found to suppress matrix metalloproteinase- 9 (MMP- 9), highlighting a distinct anti-invasive mechanism (Bajpai et al. 2018). Moreover, its effect on HCT- 15 human colon carcinoma cells has been characterized by S-phase arrest (Koide et al. 1997). Notably, alizarin has demonstrated a degree of selectivity toward malignant cells, exerting a significantly lower inhibitory impact on normal cells, reinforcing its potential as a targeted anticancer agent (Fotia et al. 2012; Huang et al. 2017a, b). These findings suggest that alizarin possesses a multifaceted anticancer potential; that is why our research is aiming to investigate alizarin’s mechanism in vivo for the first time, expanding the scope to additional cancer possible targeted pathways which could strengthen the mechanistic understanding of alizarin’s anticancer effects on ERα mediated by MDM2/p-Rb/E2F1 signaling cascade and apoptosis.

## Material and methods

### Materials

#### Chromatographic materials

TLC: aluminum-backed plates were pre-coated with silica gel F_254_ (20 × 20 cm, 200 µm, 60 Å, Merck^®^, Germany). Silica gel 60/230–400 µm mesh was used for column chromatography (Whatmann^®^, UK).

#### Solvents for extraction

Chloroform, n-hexane, ethyl acetate, ethanol, and methanol were subjected to distillation before use for chromatographic purposes.

### NMR spectrophotometer

1D ^1^H-NMR & ^13^C NMR spectra (chemical shifts in part per million (ppm) and coupling constants in Hz) were recorded on Bruker Avance DRX 400–600 MHz spectrometers using DMSO-d_*6*_ as solvents, using the residual solvent signal as an internal standard.

### Plant material

The identity of the *Rubia tinctorum* roots was established in the Faculty of Science at Suez Canal University after they were purchased from an Egyptian market. A sample with the code 2019-RT was submitted to Pharmacognosy Department, Faculty of Pharmacy, Suez Canal University.

### Extraction of the plant material

Two kilograms of the roots was pulverized, dried, and extracted with methanol using. A rotary evaporator was used to dry the extract under vacuum, yielding 250 g of brownish-red *Rubia tinctorum* methanolic extract (RT-M).

### Fractionation of the crude extract

Using 180 g of weight suspended in 4 L of water, four primary fractions—for *Rubia tinctorum* Hexane (RT-H), *Rubia tinctorum* chloroform (RT-C), *Rubia tinctorum* ethyl acetate (RT-E), and *Rubia tinctorum* butanol (RT-B)—were obtained by repeated extractions with *n*-hexane, chloroform, EtOAc, and *n*-BuOH. The four extracts were preserved for purification and concentrated under reduced pressure.

### Isolation of alizarin from the chloroform extract

A portion of 18 g of the chloroform extract was chromatographed on a silica gel column packed in chloroform, and the elution was done progressively starting with 100% chloroform and working up to (CHCl_3_: MeOH = 95:5). TLC monitored the fractions in order to gather comparable results. From the chloroform extract, 14 fractions (RT-T1 through RT-T14) were recovered.

### Isolation of alizarin from fraction 6

The effluent was collected in fractions after fraction 6 (RT-T 6) was subjected to a silica gel column and eluted with gradients of CHCl_3_/MeOH starting with 100% CHCl_3_ and increasing in polarity to 85:25. Similar elutes were combined to afford 3 sub-fractions (RT-T6 - 1 ~ RT-T6 - 3). After that RT-T6 - 1 was subjected to silica gel column chromatography using (n-hexane: EtOAc = 9:1) yielding two sub-fractions (RT-T6 - 1-V1 and RT-T6 - 1-V2). After that, the subfraction (RT-T6 - 1-V2) was subjected to a second round of silica gel column chromatography using (n-hexane: EtOAc = 8:2) to produce pure alizarin (25 mg). So, genuine alizarin was purchased from the market in order to maintain the in vivo biological activity due to the limited isolated amount of alizarin.

### MTT assay of alizarin

#### In vitro cell culture

Cancer cells (MCF7, human breast adenocarcinoma and MDA-MB- 231, triple negative breast cancer cell line) were obtained from American Type Culture Collection (ATCC, Manassas, USA). MCF- 7 cell line was developed on Roswell Park Memorial Institute medium (RPMI 1640) supplemented with 1% of 100 mg/mL streptomycin, 100 units/mL of penicillin and 10% of heat-inactivated fetal bovine serum in a humidified, 5% (v/v) CO2 atmosphere at 37 ºC, while developing MDA-MB- 231 on Dulbecco’s modified Eagle medium (DMEM) culture media with the same previous additives and conditions.

#### Cytotoxicity assay by 3-[4,5-dimethylthiazole- 2-yl]− 2,5-diphenyltetrazolium bromide (MTT)

Growing cells from diverse cell lines were trypsinized, counted, and put at the suitable densities (5000 cells/0.33 cm^2^ well) into 96-well microtiter plates. Cells were kept in a humid air at 37 °C for 24 h. Then, different concentrations of alizarin were added, along with Tam and Dox (0, 0.1, 1,10, 100, and 1000 µM) for 48 h. The viability of treated cells was measured by the MTT technique as follow. Media were removed; cells were kept with 200 μL of 5% MTT solution/well (Sigma Aldrich, MO) for 2 h to convert the dye to a colored-insoluble formazan crystal. The residual solution was removed and the formazan crystals were dissolved in 200 μL/well acidified isopropanol for 30 min, covered with aluminum foil and with continuous shaking using a Max Q 2000 plate shaker (Thermo Fisher Scientific Inc., MI) at room temperature. Absorbance was measured at 570 nm using Epoch- 2 plate reader (Bio-Tek, USA). The cell viability was expressed as percentage of control, and the concentrations that prompts 50% of maximum inhibition of cell proliferation (IC50) were determined using Graph Pad Prism version 9 software (Graph Pad software Inc., CA) (Mosmann 1983; Scudiero et al. 1988).

### In vivo study

Ehrlich solid tumor (EST) is a murine mammary adenocarcinoma model that has been widely used as a breast cancer research model. The effect of alizarin against EST via ERα/MDM2/p-RB/E2F1 axis and its apoptotic effect were investigated (Elhady et al. 2022).

#### Tumor induction

Ehrlich ascites carcinoma (EAC) cells were obtained from the Tumor Biology Department of Cairo University’s National Cancer Institute. EAC was used to create a solid tumor. A suspension of EAC cells (2.5 × 10^6^ cells/100 μL) in normal saline was prepared. The cells were counted under the microscope by a hemocytometer. After shaving, the mice were injected subcutaneously with a 100 μL EAC suspension in the lower ventral side (Lazarus 1966).

#### Dose selection

A preliminary study was conducted with two dose levels 50 and 100 mg/kg and both went with significant outcomes, the selection of these exact dose levels was based on a structurally similar anthraquinone derivative—SZ- 685 C—with established antitumor activity against ER + breast cancer. This marine-derived anthraquinone, previously tested in xenografted MCF- 7 tumors in female mice, was administered at 50 mg/kg, and this dose was successful to reduce tumor weight and size (Zhu et al. 2012). The larger dose was selected based on a structurally analogous anthraquinone, emodin which has demonstrated anticancer qualities in various in vitro and in vivo studies, including breast cancer models. However, at 40 mg/kg, it failed to significantly reduce tumor weight or size in some xenograft models. Additionally, it was used for the treatment of colon cancer with a range of doses 50–100 mg/kg. Based on this, we opted for an increased dosage of 100 mg/kg, rather than a lower dose, to enhance therapeutic efficacy (Akkol et al. 2021).

#### Study design

The study used 30 Swiss albino mice weighing 25–30 g obtained from the Egyptian Organization for Biological Products and Vaccines (Vacsera, Giza, Egypt). The mice were kept in plastic cages at a temperature of 25 °C with a normal light/dark cycle and access to water and food. The mice were left for a week to adapt before the experiments. They were distributed into three groups at random (10 mice each). Tumor cells were inoculated into all groups (control, gp1, and gp2). The first group was designated as the Ehrlich control group; the mice received (2.5 × 10^6^ cells/100 μL) of EAC at day zero (the day of tumor cell inoculation), exactly the same amount groups 2 and 3 received. Group 1 received saline (5 mL/kg/day, PO) form day 7 (beginning of the treatment) till the day of decapitation (14 days successively) which is the day 21. Groups 2 and 3 were treated by (50 mg/kg and 100 mg/kg PO) of alizarin, respectively. Groups 2 and 3 were given the treatment daily from day 7 to day 21, as summarized in Table 1. Alizarin was dissolved in Tween 80 and distilled water as a vehicle. Each mouse was receiving 0.2 mL of the treatment, for group 2 (50 mg/kg), the total daily final volume is 2 mL, since each mouse was receiving 1.25 mg/day of alizarin assuming that the average weight was 25 gm and each group contains 10 mice, so 12.5 mg alizarin was dissolved into 2 mL of the vehicle as follows: 40 μL Tween 80 (1% v/v in final volume) (Márquez 2024) was added to 12.5 mg of alizarin powder while stirring, followed by mixing for 2–3 min using a vortex mixer; the distilled water was added gradually while stirring continuously to make a total volume of 2 mL, to ensure complete homogenization; we used ultra-sonication (5–10 min) or magnetic stirring (15 min) to form a stable suspension. In Group 3, the experimental procedures were consistent with the prior group, with the exception that the alizarin dosage was increased to 100 mg/kg, necessitating a total administration of 25 mg. There were no precautions for storage as the drug was freshly prepared on a daily basis. The in vivo study followed *the Guide of the Care and Use of Laboratory Animals*. The ethical committee of Suez Canal University’s Faculty of Pharmacy approved the study protocol (202011MA2).

**Table 1:** Summary of the study design

Group	Treatment
Ehrlich control group	Received saline (5 mL/kg/day, PO) form day 7 (beginning of the treatment) till the day of decapitation (14 days successively)
Group (2)	Received alizarin (50 mg/kg/day; PO) form day 7 (beginning of the treatment) till the day of decapitation (14 days successively)
Group (3)	Received alizarin (100 mg/kg/day; PO) form day 7 (beginning of the treatment) till the day of decapitation (14 days successively)

#### Sample collection

After the mice were anaesthetized with thiopental sodium (50 mg/kg, IP) on day 21 following EAC inoculation, by which time it had transformed into EST; blood samples were taken from the orbital sinus (retro orbital plexus). After leaving the samples for 20 min, the serum was separated by centrifugation at 2000 RPM for 15 min. The tumor masses were meticulously excised using a sterile dissection with scalpels, ensuring minimal tissue disruption. Subsequently, the tumor discs were carefully isolated, followed by precise weighing using an electronic four dismal’s balance to ensure accurate measurement of tumor weight. Each tumor sample was then sectioned into two portions; one portion of each tumor disc was fixed in 10% neutral buffered formalin for immunohistochemical studies, while the other portion was kept at − 80 °C for PCR and western blot analysis. The tumor size was calculated by the following formula: [Length (cm) × Width^2^ (cm)]**/**2 (Ellethy 2019).

#### Immunohistochemical assessment of the expression of ER-α and apoptotic markers in the tumor tissue

The tumor discs were placed overnight in 10% neutral buffered formalin, followed by embedding in paraffin. Then, deparaffinization was performed. Antigen retrieval was made according to the Tris/EDTA buffer (pH = 9) antigen retrieval protocol. Staining was performed using the EnVision™ FLEX HRP-labeled high-pH method in accordance with the manufacturer's instructions (Dako, Glostrup, Denmark). Primary polyclonal antibodies for p53 (bs- 0033R), Bax (bs- 0127R), and ERα (bsm- 53007 M) (Bioss Inc., Woburn, MA, USA) were diluted in PBS (normal phosphate buffered saline) at a ratio of 1:250. Mayer’s hematoxylin was then used as a counterstain. The semiquantitative analysis of the immunohistochemical reactions was done using ImageJ. The photos were taken with a camera (HDCE30 C) using its software which was connected to an optical microscope (Optika B352 A, OPTICA, Via Rigla, Italy); the images were quantified with the ImageJ MacBiophotonics (National Institutes of Health, USA) software package developed by McMaster University (Ontario, Canada). Using the color deconvolution plugin, the expressions of p53, Bax, and ERα were all evaluated, and the percentages of stained regions were computed.

#### Assessment of MDM2/E2 F1/P-RB expression by western blot analysis

Cells were lysed with NP- 40 lysis buffer (50 mMTris, pH 7.4; 150 mMNaCl; 1% NP- 40). Proteins from the whole cell lysate were separated in a 12% SDS PAGE. The electrophoresed proteins were blotted onto Amersham™ Hybond^®^ P (GE10600021, SIGMA) Western blotting membranes, PVDF and incubated with the primary antibodies; mouse anti-β-actin (1:1000), mouse anti-MDM2 (1:500), mouse anti-E2 F- 1 (1:500), and mouse anti-p-Rb (1:500) at 4 °C overnight at room temperature for 1 h. The primary antibodies against MDM2 (SMP14): sc- 965, E2 F- 1 (KH95): sc- 251 and p-Rb (E- 10): sc- 271930 purchased from Santa Cruz Biotechnology, Santa Cruz, CA, USA, were used while antiactin used as the loading control. Quantification of the western blot bands was performed via Image analysis software on the ChemiDoc MP imaging system (version 3) formed by Bio-Rad (Hercules, CA). Relative density of each band was evaluated and normalized with β-actin.

#### Quantitative real‐time PCR (qRT‐PCR) for assessment of the expression of caspase 3 in tumor tissue

Following the manufacturer’s instructions, an SV total RNA isolation kit (Promega, Madison, USA) was used to separate the total RNA from the tumor tissue. At − 80 °C, the isolated RNA was kept. The concentration and purity of the extracted RNA were measured by a NanoDrop spectrophotometer (Thermo Fisher Scientific, USA). A GoTaq^®^ 1‐Step RT‐qPCR System (Promega, Madison, WI, USA) was used to evaluate the expression of *Casp3* gene. β‐Actin was designated as the housekeeping gene, and it was amplified using two primers:

- Forward: CCTCAGAGAGACATTCATGG
- Reverse: GCAGTAGTCGCCTCTGAAG

The reaction was implemented in a StepOnePlus™ Real‐Time PCR thermal cycler (Applied Biosystems, Waltham, MA, USA). 20 μL mixture of each sample consisted of 4 μL of the RNA template, 1 μL of each of the forward and reverse primers, 0.4 μL of GoScript™ reverse transcriptase (RT) mix for 1‐step RT‐qPCR, 10 μL of GoTaq^®^ qPCR master mix, 0.31 μL of supplemental CXR reference dye, and 3.29 μL of nuclease‐free water. Reverse transcription was performed for 15 min at 37 °C followed by inactivation of reverse transcriptase enzyme. The primary denaturation was employed for 10 min at 95 °C. Next, 40 cycles of denaturation at 95 °C for 10 s annealing at 52 °C for 30 s, followed by extension at 72 °C for 30 s, the cycle’s threshold (Ct) value was recorded, and the ΔCt was calculated against β‐actin. Each sample’s fold change was calculated.

#### Statistical analysis

The data were analyzed statistically using the statistical version 18 of the SPSS software computer package (SPSS Inc, USA). Mean ± standard deviation (SD) was used to determine the values of the parameters expressed. One-way ANOVA was used to perform comparisons, and then a Bonferroni post hoc test for multiple comparisons. Differences were considered statistically significant at *p* < 0.05.

### Docking study

Two different molecular docking studies (Gaber et al. 2025) were carried out using Discovery Studio (Jejurikar and Rohane 2021) and PyMol (Yuan et al. 2017) to evaluate the binding affinity of the examined compound (alizarin) toward both the core binding pocket within the ligand-binding domain (LBD) of the estrogen receptor and the second site on its surface. The estrogen antagonist 4-hydroxytamoxifen (HT) co-crystallized at the two aforementioned pockets was inserted as a reference standard.

First, the chemical structure of the investigated compound (alizarin) was obtained from the PubChem website and prepared by energy minimization and partial charges optimization (Aljuhani et al. 2022; Salem et al. 2022). On the other hand, the X-ray structure of the target estrogen receptor was downloaded from the Protein Data Bank (ID: 2 FSZ) (Wang et al. 2006) and prepared for docking by three dimensional hydrogenation, correction and energy minimization as well (Aziz et al. 2021; Shaaban et al. 2023).

Finally, two different molecular docking processes were performed to evaluate the binding affinity of alizarin at both the core binding pocket within the LBD of the estrogen receptor and the second site on its surface. The best pose for alizarin in each docking process (depending on the score, root mean square deviation (RMSD), and binding mode criteria) was selected for further investigations (Kutkat et al. 2022).

## Results

### Identification of the isolated compound from chloroform fraction

**Fig. 1:**
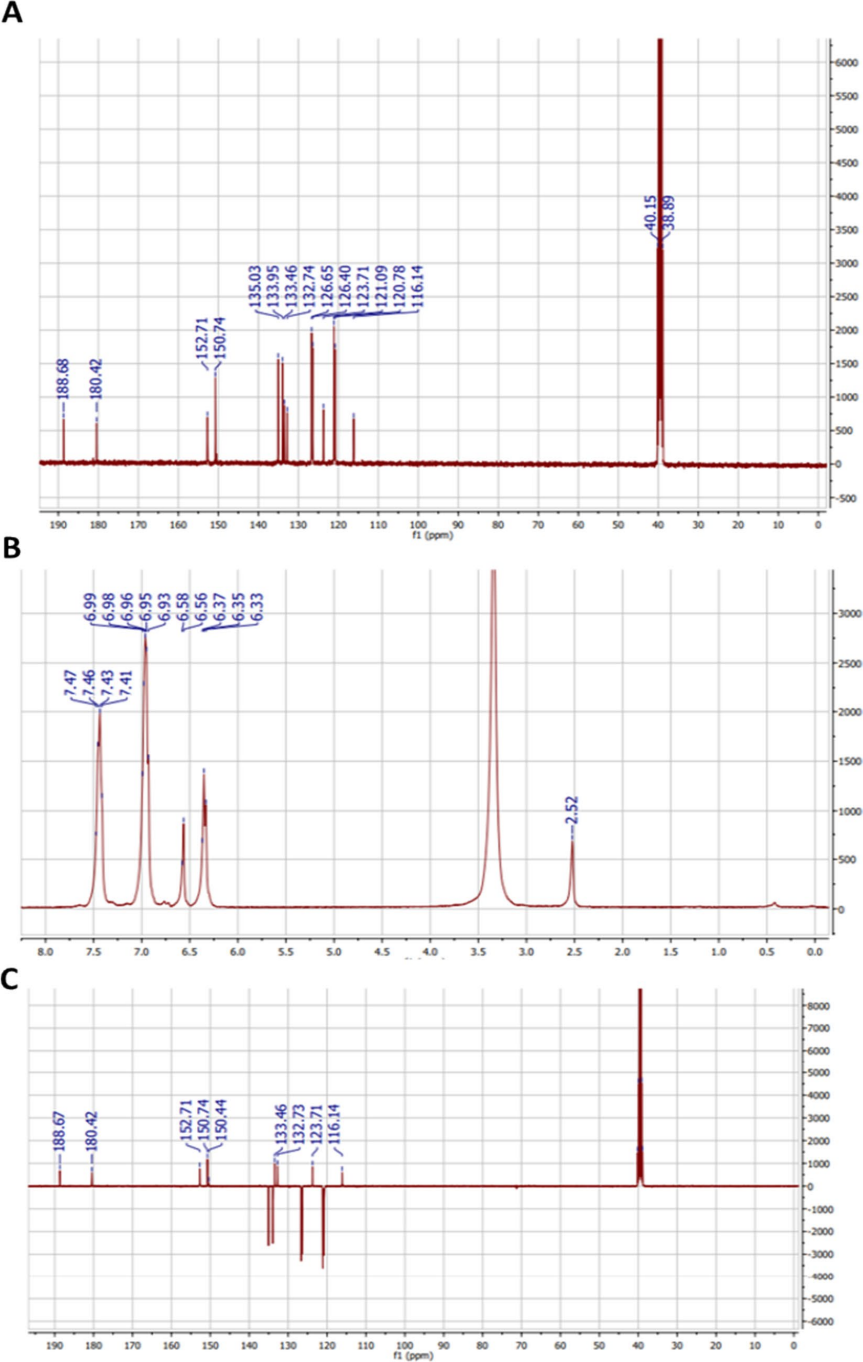
**A** ^1^H-NMR spectrum of Alizarin in (DMSO-*d*_6_, 400 MHz). **B** ^13^C-NMR spectrum of Alizarin in (DMSO-*d*_6_, 100 MHz). **C** APT spectrum of Alizarin in (DMSO-*d*_6_, 100 MHz)

In spectroscopic analysis, ^1^H-NMR (400 MHz) and ^13^C-NMR (100 MHz) data for alizarin in DMSO − *d*_6_ are listed in Table 2 and Fig. 1.

**Table 2:** 1H (400 MHz) and 13C (100 MHz) NMR spectroscopic data of compound (1) (DMSO‐d6, J in Hz, and δ in ppm) compared with literature

No	Compound 1		Alizarin (Siddiqui et al. 2007)	
	*δ*_C_	*δ*_H_	*δ*_C_	*δ*_H_
–1	152.7	–	152.8	–
2	150.7	–	150.7	–
3	121.0	6.35 (d, *J* = 8)	125.8	7.13 (d, 8.0)
4	120.7	6.58 (d, *J* = 8)	121.0	7.71 (d, 8.0)
5	126.7	7.41–7.47 (m)	126.5	8.21 (br.d, 7.7)
6	135.0	6.93–6.99 (m)	135.0	7.93 (br.dd, 7.7)
7	134.0		133.8	
8	126.4	7.41–7.47 (m)	126.4	8.20 (br.d, 7.7)
9	188.6	–	188.6	–
10	180.4	–	180.3	–
11	132.7	–	132.6	–
12	133.5	–	133.5	–
13	116.1	–	116.1	–
14	123.7	–	123.6	–

### MTT assay of isolated alizarin

**Fig. 2:**
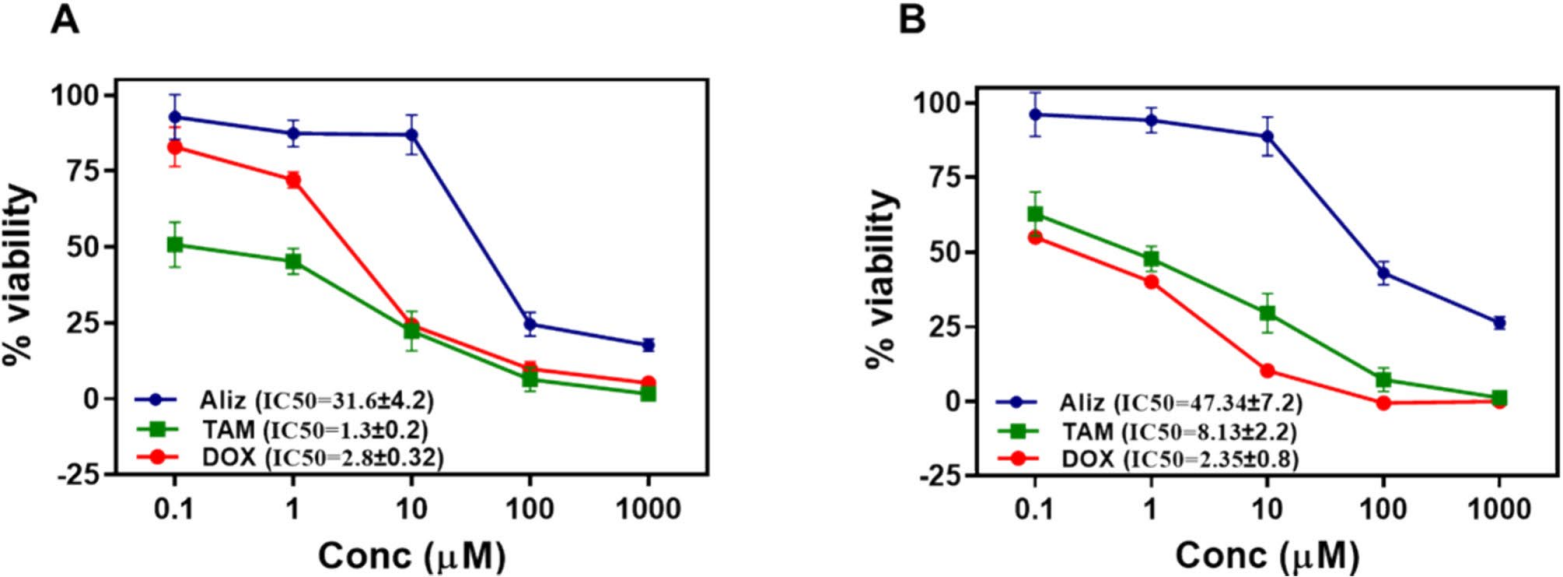
Effect of alizarin, tamoxifen, and doxorubicin on the viability of **A** MCF-7 and **B** MDA-MB- 231

In the view of the obtained data, alizarin showed cell viability against the MCF‐7 cell line with IC50 of 31.6 in comparison with tamoxifen as a positive control with IC50 1.3. Alizarin significantly reduced growth of the MCF- 7 cells in a dose-dependent way expressing that alizarin has a promising cytotoxicity. Moreover, alizarin presented cell viability against the MDA-MB- 231 cell line with IC50 of 47.3 when compared with tamoxifen with IC50 8.13. However, it is worthy to note that tamoxifen is a prodrug that requires conversion to its active metabolite, endoxifen, via cytochrome P450 family 2D6 (CYP2D6), to exert its primary therapeutic effects. Tamoxifen itself has weak binding affinity to estrogen receptors and limited antiestrogenic activity in its unmetabolized form. Consequently, in vitro results using tamoxifen may not accurately reflect its in vivo efficacy, as the metabolic activation process is absent in cell culture systems. That is why it was important to use doxorubicin as another positive control (Fig. 2).

### In vivo antitumor effects of isolated alizarin

#### Effect of alizarin on tumor weight and volume

**Fig. 3:**
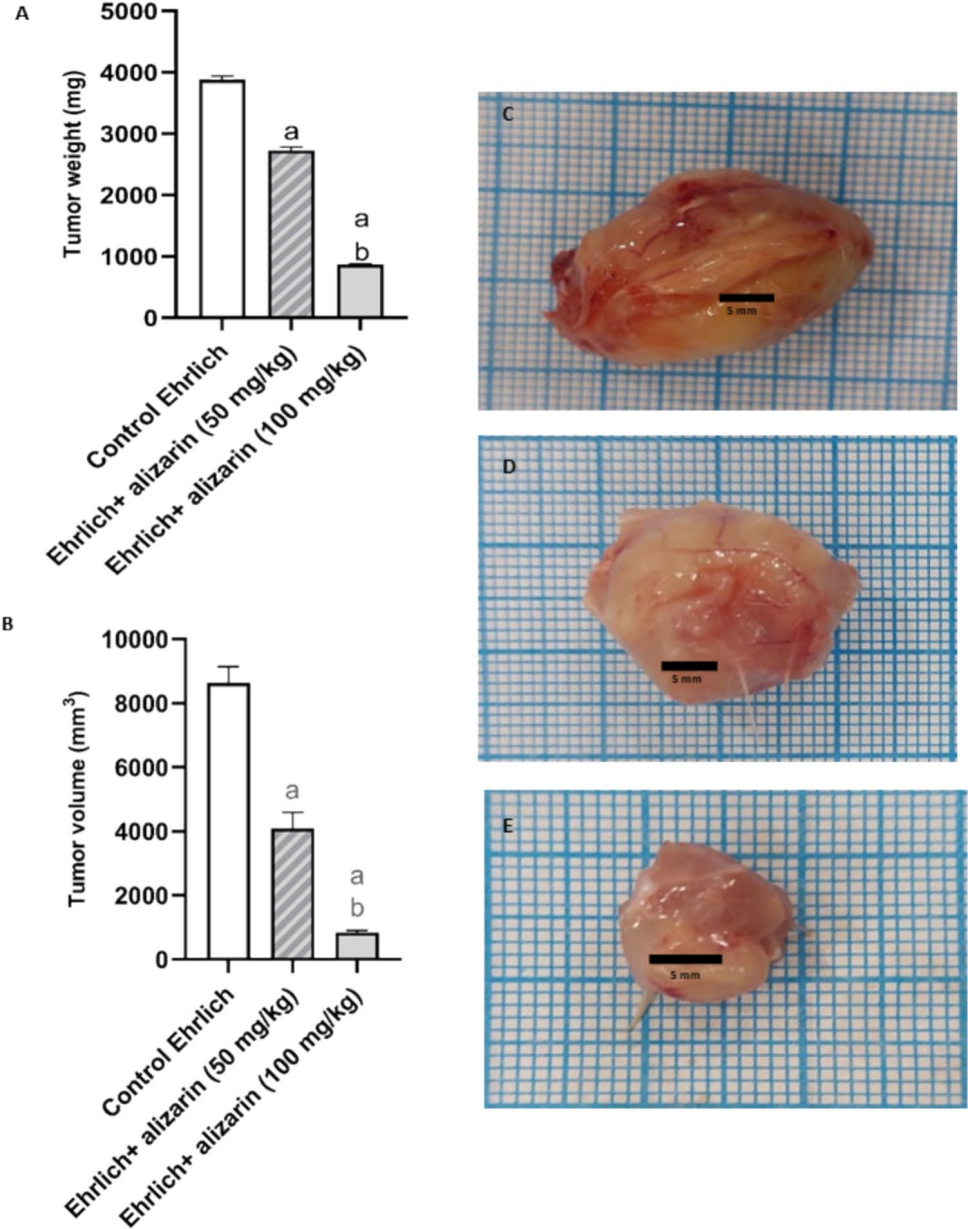
Effect of alizarin (50 mg/kg and 100 mg/kg) on tumor weight compared to Ehrlich control group (**A**). Effect of alizarin (50 mg/kg and 100 mg/kg) on tumor volume compared to Ehrlich control group (**B**). Representative photograph for Ehrlich control group (**C**). Representative photograph for group treated with (50 mg/kg) alizarin (**D**). Representative photograph for group treated with (100 mg/kg) alizarin (**E**). All data expressed as mean ± SD. All data were analyzed using ANOVA followed by a Bonferroni post hoc test. (a) Significant compared with the control group at *p* < 0.05. (b) Significant compared with the small dose group at *p* < 0.05

Groups treated with alizarin at two dose levels (50 and 100 mg/kg) presented a significant (*p* < 0.05) decrease in tumor weight and volume when compared to Ehrlich control group in a dose dependent manner as shown in Fig. 3.

#### Effect of alizarin on ER-α

**Fig. 4:**
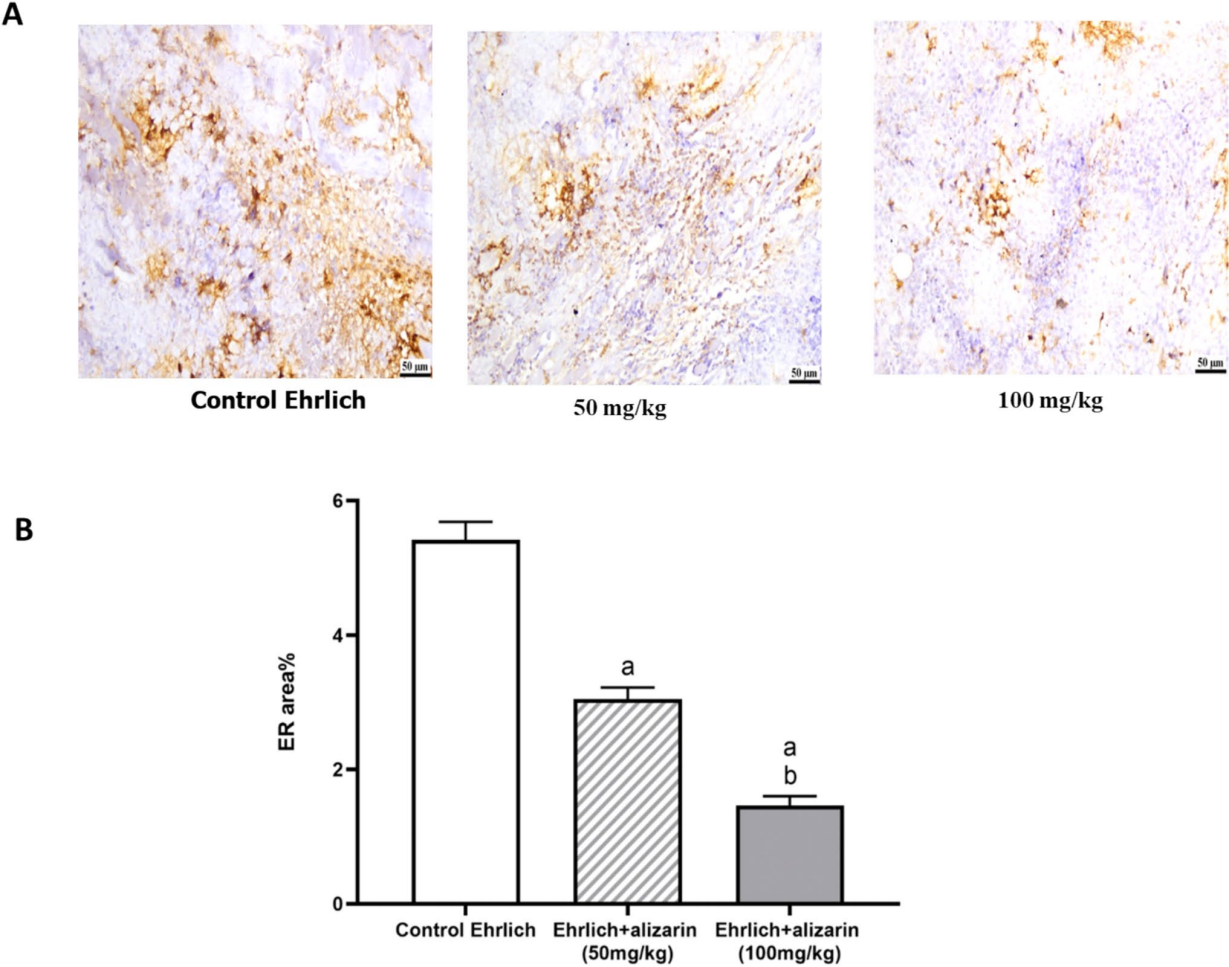
Effect of alizarin (50 mg/kg and 100 mg/kg) on ERα expression in different experimental groups. **A** Representative photomicrographs of ERα. **B** The percentage of positive immunohistochemical reaction (brown stained area) analyzed by ImageJ software. All data expressed as mean ± SD. All data were analyzed using ANOVA followed by a Bonferroni post hoc test. (a) Significantly different compared with the control group at *p* < 0.05. (b) Significantly different compared with the small dose group at *p* < 0.05

Immunohistochemical staining of ERα revealed high expression of ERα in Ehrlich control group. Interestingly, data revealed significant (*p* < 0.05) downregulation of ERα in alizarin treated groups (50 and 100 mg/kg) compared to the Ehrlich control group (Fig. 4A, B).

#### Effect of alizarin on MDM2/p-Rb/E2F1 expression in the tumor cells

**Fig. 5:**
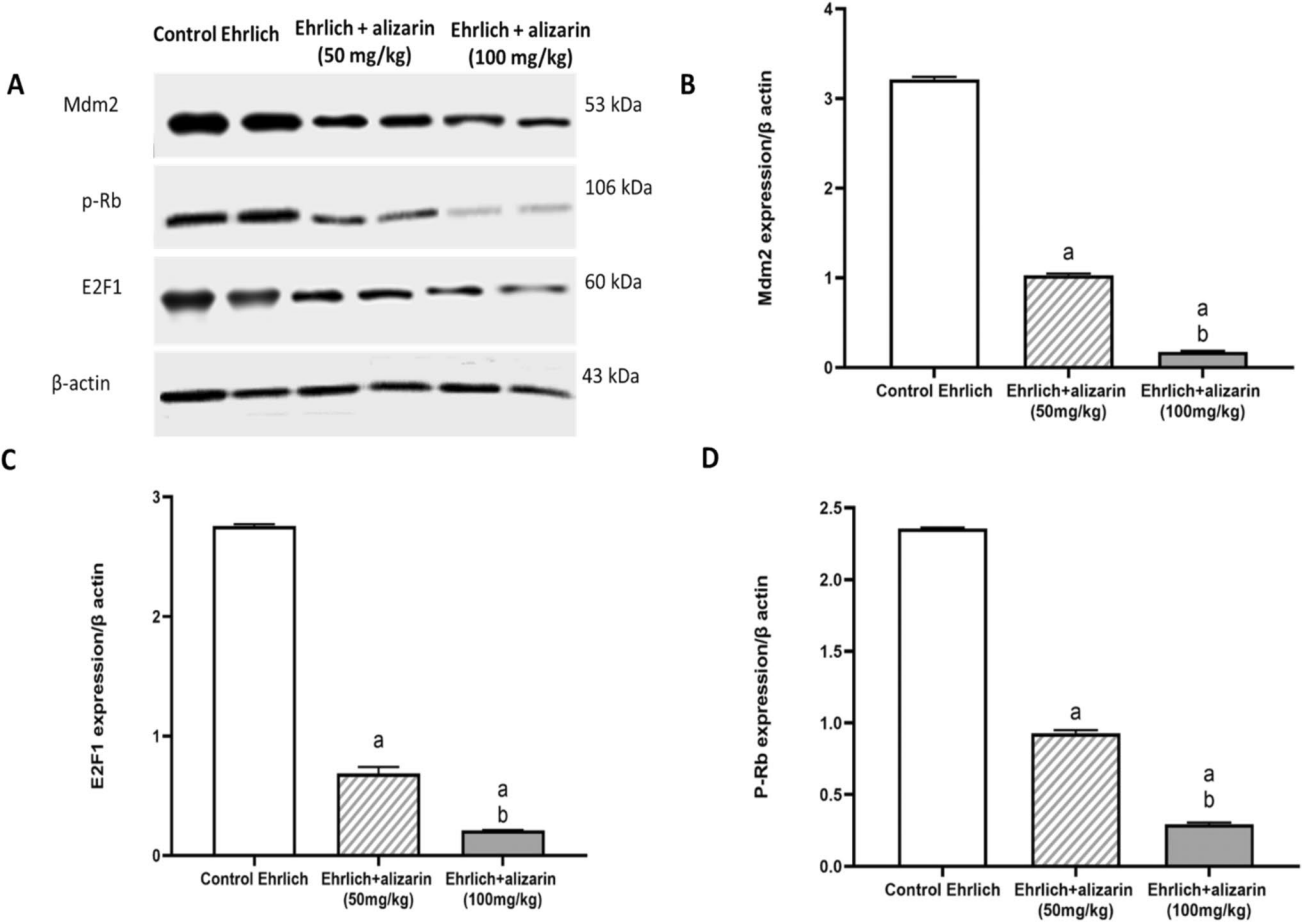
Effect of alizarin (50 mg/kg and 100 mg/kg) on MDM2/p-Rb/E2F1 axis. **A** Western blot analysis showing protein expression of MDM2, p-Rb, E2 F1 and β-actin in tumor tissues. **B** Quantified data of MDM2 normalized to β-actin. **C** Quantified data of E2F1 normalized to β-actin. **D** Quantified data of p-Rb normalized to β-actin. Results are expressed as mean ± SD. All data were analyzed using ANOVA followed by a Bonferroni post hoc test. (a) Significantly different compared with the control group at *p* < 0.05. (b) Significantly different compared with the small dose group at *p* < 0.05

The MDM2 is mainly overexpressed by estrogen signals, therefore MDM2 expression was measured to examine the effect of alizarin on estrogen signaling pathway. Elevated MDM2 expression was detected in control Ehrlich group. On the contrary, alizarin treated groups displayed significant (*p* < 0.05) decrease in the MDM2 expression in a dose dependent manner compared to the Ehrlich control group (Fig. 5B**)**.

The E2 F1 is identified to stimulate cell cycle progress. Upon examination of E2 F1 expression in different groups, strong expression of E2 F1 protein was observed in control Ehrlich group. While, treatment with alizarin decreased expression of E2 F1 in tumor cells significantly (*p* < 0.05) compared to control Ehrlich group in a dose dependent way (Fig. 5C).

Phosphorylated Rb reduces the negative regulatory effect of sp1 (the promoter specificity factor) by interacting with MDM2. Ehrlich control group exhibited robust expression of p-Rb protein. Treatment with alizarin reduced p-Rb expression significantly (*p* < 0.05) compared to control Ehrlich group in a dose-dependent manner (Fig. 5D).

#### Effect of alizarin on expression of P53 and BAX in tumor cells

**Fig. 6:**
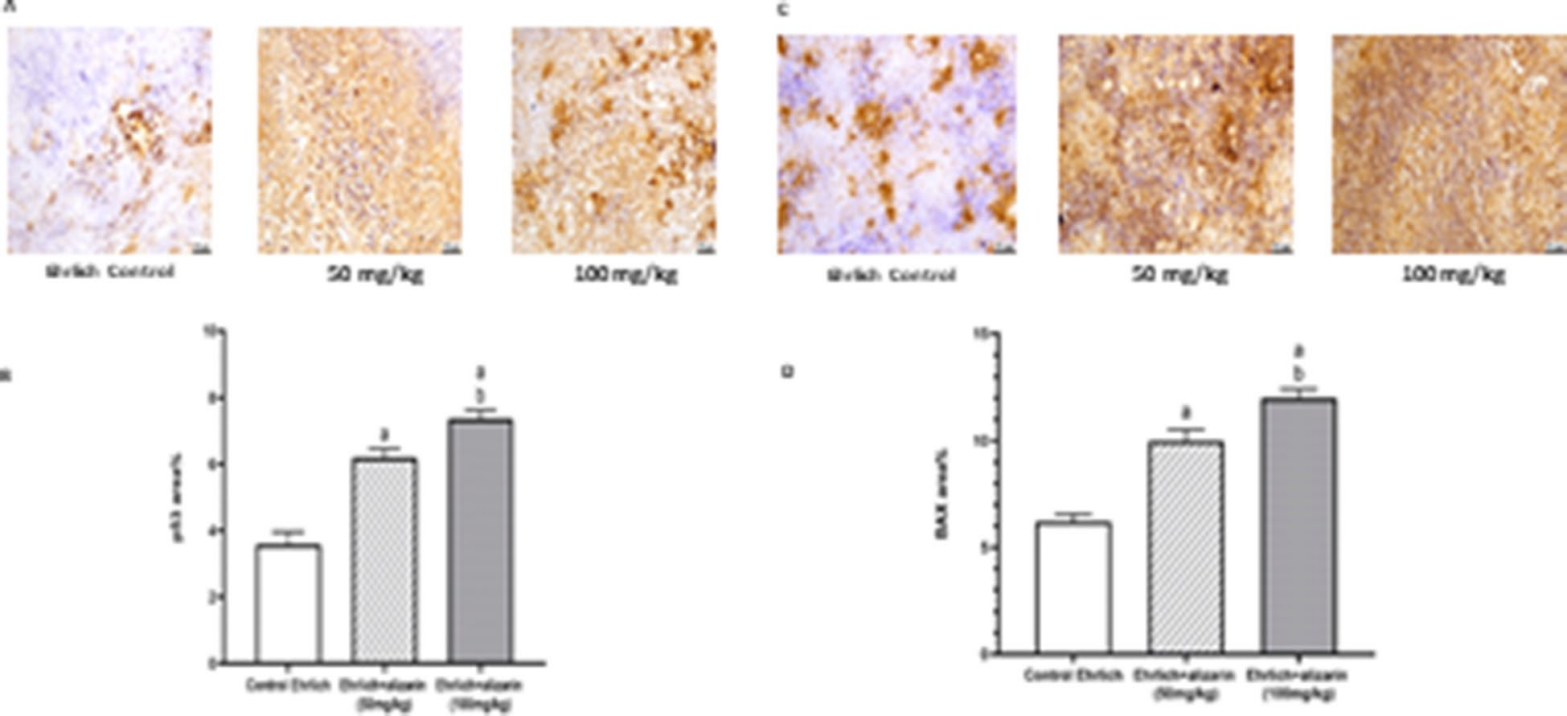
Effect of alizarin (50 and 100 mg/kg) on p53 & BAX expression in different experimental groups. **A, C** Representative photomicrographs of p53 & BAX respectively. **B, D** The percentage of positive immunohistochemical reaction of p53 & BAX respectively (brown stained area) analyzed by Image J software. All data expressed as mean ± SD. All data were analyzed using ANOVA followed by a Bonferroni post hoc test. (a) Significantly different compared with the control group at *p* < 0.05. (b) Significantly different compared with the small dose group at *p* < 0.05

The control Ehrlich group displayed marked reduced expression of p53 in the proliferating tumor cells. Meanwhile, p53 expression was increased in gp1 and gp2 in a dose-dependent manner. Statistical analysis of p53 area % revealed a significant (*p* < 0.05) rise in the treated groups when compared with the control Ehrlich group (Fig. 6A, B).

Slight immune staining for Bax was noticed in neoplastic cells of the control Ehrlich group. However, alizarin-treated groups demonstrated increased positive expression of Bax tumor cells compared to control Ehrlich group. Statistical analysis of Bax area % expression revealed a significant increase in gp2 (*p* < 0.05) when compared with other groups (Fig. 6C, D).

#### Effect of alizarin on caspase 3 expression in tumor cells

**Fig. 7:**
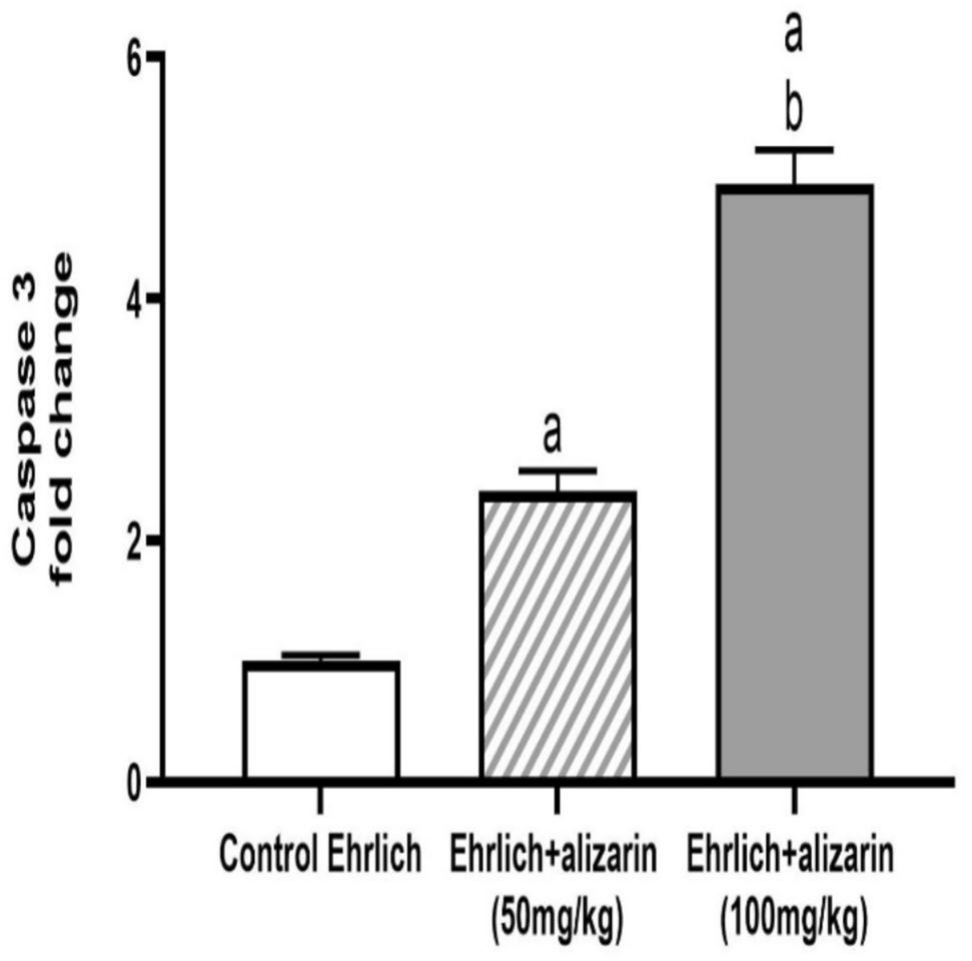
Effect of alizarin (50 and 100 mg/kg) on caspase 3 expression in different experimental groups. All data expressed as mean fold change ± SD. All data were analyzed using ANOVA followed by a Bonferroni post hoc test. (a) Significantly different compared with the control group at *p* < 0.05. (b) Significantly different compared with the small dose group at *p* < 0.05

Since caspase 3 is a crucial component of the apoptosis process, its expression was investigated. The gene expression of caspase 3 was significantly (*p* < 0.05) raised in the treatment groups compared with the control Ehrlich group indicating dose dependent apoptotic activity of alizarin (Fig. 7).

### Molecular docking

**Fig. 8:**
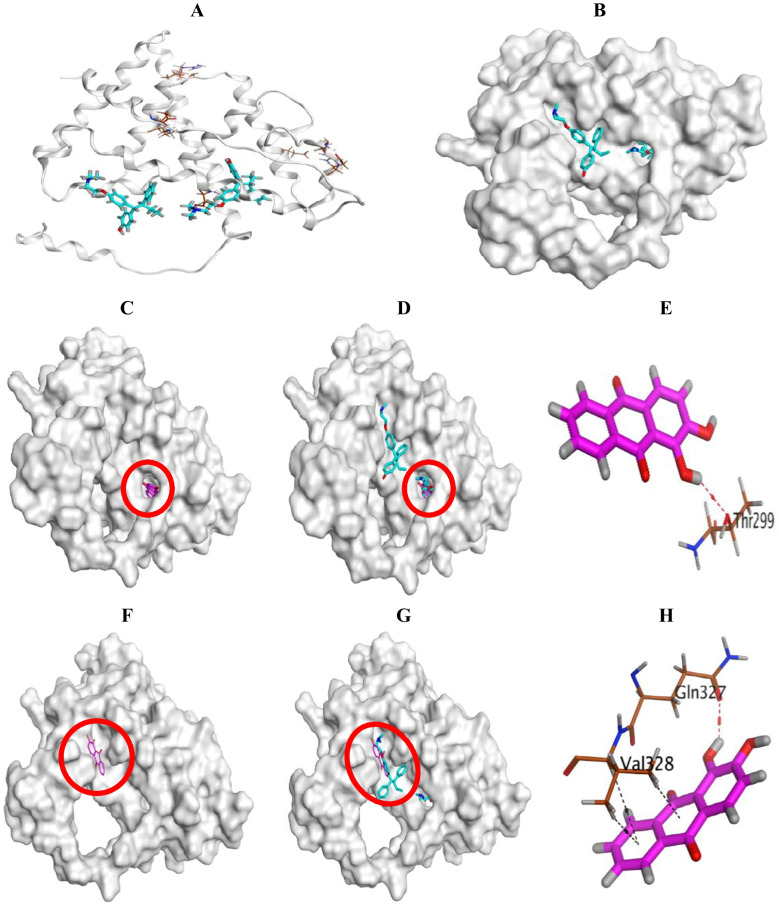
3D binding interactions, and 3D positioning of alizarin inside both the core binding pocket (LBD) of estrogen receptor (**A**) and the second site on its surface (**B**). Alizarin alone in the LBD (**C**); Superimposition of alizarin and the co-crystallized HT antagonist in the LBD (**D**); Binding interactions of alizarin inside the LBD (**E**); Alizarin alone in the surface pocket (**F**); Superimposition of alizarin and the co-crystallized HT antagonist in the surface receptor (**G**); Binding interactions of alizarin inside the surface receptor (**H**)

The downloaded estrogen receptor (PDB ID: 2 FSZ) was visualized and showed the presence of two pockets within the target receptor. The first pocket observed in the core is known as the ligand-binding domain (LBD) and is occupied by one molecule of the estrogen antagonist 4-hydroxytamoxifen (HT). Notably, HT got stabilized through the formation of three H-bonds with GLU305, ASP303, and LYS480, besides a pi-H bond with MET479. Instead, the second pocket was found on the surface of the estrogen receptor and occupied by a second molecule of HT. The second HT antagonist formed three H-bonds with GLN327 (2) and ALA313, in addition to a pi-H bond with VAL328 (Fig. 8A, B).

The first docking process for the prepared database of both alizarin and HT was carried out at the first core pocket of the estrogen receptor (LBD). Notably, alizarin was found to get deeper inside the LBD than the co-crystallized HT antagonist as shown in Fig. 8C, D. Alizarin achieved a binding score of − 5.56 kcal/mol (RMSD = 1.34 Å). It showed the formation of one H-bond with THR299 amino acid (Fig. 8E). Therefore, alizarin only formed one H-bond which was enough to stabilize it deeper inside the core pocket with the aid of its smaller size compared to the HT antagonist.

However, the second docking process showed the presence of alizarin inside the minor cleft of the second surface estrogen receptor (Fig. 8F, G). It was able to record a binding score of − 4.57 kcal/mol (RMSD = 2.02 Å) with the formation of one H-bond with GLN327. Also, it bound VAL328 with three pi-H interactions (Fig. 8H).

## Discussion

Several studies were (Kundu et al. 2017; Harakandi et al. 2021; Al-Hussaniy and Al-Zobaidy 2024) conducted on different cell lines to examine the role of MDM2 and p53 in cancer but the relation between them is still controversial. The contribution of ERα in proliferation of breast cancer cells and its effect on MDM2 signaling has attracted attention in research (Bianco et al. 2022, Wege et al. 2022). Consequently, the present research was performed to study the antiestrogenic effect of different doses of alizarin and its effect on MDM2/Rb/E2F1 pathway and apoptosis.

Breast cancer-mediated cell proliferation is fueled by estrogen. ERα is a nuclear hormone receptor and oncoprotein which is found in nearly 70% of breast tumors (Karami Fath et al. 2022). Estrogen receptor positively controls growing and development of numerous tissues (Wang et al. 2021). MDM2 oncogene is expressed at many levels according to cell type and condition. MDM2 transcription and its protein levels are both enhanced in ER + cells exposed to estrogen (Maiuri et al. 2010). Notably, MDM2 expression exhibits a positive correlation with ERα expression in human breast tumors and ERα-positive breast cancer cell lines. Furthermore, ERα has been implicated in the upregulation of MDM2 expression (Ghavidel et al. 2021, Gözen 2021, Yao et al. 2024).

Estrogen stimulates cell cycle progress by endorsing the G1 to S phase transition and Rb phosphorylation (Mir and Jan 2023). Rb is a tumor suppressor protein that regulates E2 F1 inhibition of DNA replication. RB binds to the activation domain of E2 F1 and hushes it resulting in S-phase arrest. The crucial role of Rb depends on its capability to interact with the E2 F family of transcription factors to form active repressor complexes and negatively control expression of E2 F-dependent genes (Roufayel et al. 2021).

It is well established that E2 F1 is essential for cell cycle control because unregulated expression of the E2 F1 protein encourages cell entry into S phase leading to increased proliferation (Swetzig et al. 2016). MDM2 interacts with Rb and E2 F- 1, to promote cell cycle G1-S transition. MDM2 proliferative targets include the activation of E2 F1 transcriptional activity and suppression of tumor suppressive effects of Rb through binding to Rb and inhibiting the function of Rb-E2 F repressor (Wu and Wu 2021). MDM2 knockdown results in a reduction in E2 F1 protein (Swetzig et al. 2016). The oncogenic protein MDM2 is frequently expressed in high levels in breast cancer cells with wild-type p53 (Opoku et al. 2021).

One of the main purposes of active p53 is to induce apoptosis. The p53 tumor suppressor protein binds to specific DNA sequences and regulates the transcription of many genes involved in cell-cycle arrest and apoptosis, thus cell cycle arrest associated with p53 upregulation (Ghate et al. 2013).

Alizarin has been extensively investigated for its potential anticancer activity across a range of malignancies, including papilloma, as well as bone, pancreatic, hepatic, pulmonary, mammary, ovarian, and female bladder tumors. Research findings indicate that Alizarin exhibits a significant cytotoxic effect on the majority of tested cancer cell lines. (Yao et al. 2014; Huang et al. 2017a, b; Bajpai et al. 2018). However, anticancer effect for alizarin in vivo and its antitumor mechanisms weren't fully investigated.

In the current study, treatment with alizarin decreased tumor weight compared to the Ehrlich control group. These results confirmed the cytotoxicity of alizarin performed against MCF- 7. Matching with this data, alizarin has been shown to be cytotoxic to a variety of malignant cell lines, including Hep-G2 with an IC50 of 79.27 µM (Mishra et al. 2017), MDA-MB- 231 with an IC50 of 62.1 µg/mL, and U- 2 OS cells IC50 was 69.9 µg/mL (Fotia et al. 2012). Another study reported a cytotoxic effect of alizarin on MGC- 803 with an IC50 of 40.35 µM (Yao et al. 2014; Huang et al. 2017a, b), concluding that alizarin is a potent cytotoxic agent especially on ERα positive breast cancer and bone cancer (Mishra et al. 2017).

It was reported that estrogen signals lead to overexpression of MDM2 triggering phosphorylation of Rb and increasing the levels of E2 F1. Interestingly, treatment with alizarin markedly decreased the expression of ERα along with downregulation of MDM2/E2 F1/p-Rb pathway in a dose dependent manner confirming the antiestrogenic effect of alizarin. This supports the idea that estrogen-MDM2 stimulates activation of the E2 F1 pathway. MDM2 overexpression is directly related to ERα overexpression and increasing the phosphorylation of Rb. Constitutive MDM2 knockdown resulted in a significant reduction in the estrogen-driven phosphorylation of Rb (Kundu et al. 2017). Similarly, ICI- 182780 (Fulvestrant) blocked the activation signals of estrogen causing inhibition of Rb phosphorylation with subsequent G1 arrest in human breast cancer cell line MCF- 7 (Lin et al. 2019).

The above described cascade was typically observed in the current study implying that alizarin is interfering with proliferative signaling through ERα, hence affecting estrogenic stimulation of the tumor cells (Huang et al. 2013). ERα signaling activates cell cycle-related cyclin, CDK, and Rb genes resulting in MCF- 7 cell proliferation (Liao et al. 2014). Emodin and aloe-emodin, structurally similar anthraquinone derivatives to alizarin, were reported to have inhibitory effects on ERα (Huang et al. 2013).

Similarly, it was reported that the ERα-MDM2-Rb-E2 F1 axis was blocked in the presence of estrogen receptor antagonist fulvestrant. Fulvestrant blocks estrogen-dependent increase in MDM2 by accelerating MDM2 protein turnover (Dolfi et al. 2014). This idea was supported by data about MDA-MB- 231, triple negative breast cancer cell line, that Fulvestrant has no MDM2 effect on proliferation in the absence of estrogen receptor activation (Kundu et al. 2017), which confirms that alizarin mainly works by cutting off estrogenic signals.

It was evidenced in a study made on MCF- 7 cells treated with estradiol, that cyclin dependent kinase 2 and cyclin E-dependent kinase activity increased, along with hyperphosphorylation of Rb and S-phase entry (Kundu et al. 2017). However, upon administration of ICI 182,780, the pure antiestrogen this pathway was inhibited. It has been postulated that antagonizing ER results in inhibition of cyclin dependent kinase (CDK)/cyclin activity and the hypophosphorylation of RB to suppress transcription of E2 F-regulated genes and inhibit progression to S-phase (Pancholi et al. 2020). This explanation was very compatible with results observed in the current study explaining the antiestrogenic effects of alizarin.

In the current study, the Ehrlich control group presented weak expression of p53, Bax, and caspase 3. P53 is an important tumor suppressor gene, and it seems to have an extremely tangled relationship to ER, MDM2, E2 F, Rb, Bax, caspase3, and a lot of other cell regulatory and apoptotic related markers (Buchwald 2011, McCurdy 2016, Bianco et al. 2022, Huang et al. 2024).

It has been proved that MDM2 may regulate normal and neoplastic cell growth by inhibiting p53 function (Koo et al. 2022). It worth mentioning that p53 was shown to be in a tertiary complex with ERα and MDM2 (Liu et al. 2000, Huang et al. 2024).

A previous study revealed that AQ- 101 downregulates MDM2 and activates p53, which might be explained by ultimately inducing MDM2 degradation via binding to MDM2 and preventing its interaction with MDM4. This can be explained as co-expression of MDM2 and MDM4 repressed self-ubiquitination of MDM2 but increased p53 ubiquitination. However, doxorubicin and nutlin- 3 both stimulate p53, either by causing DNA damage or by blocking the interaction between p53 and MDM2 (Gu et al. 2018). Moreover, research on Nutlins cis imidazoline analogues, powerful MDM2 inhibitors, showed that they bind closely into the p53 pocket of MDM2, stimulating the p53 pathway, and cause apoptosis in tumor cells with wild-type p53 (Fang et al. 2020). RITA, an inhibitor of p53-MDM2 interactions binds to p53 rather than the MDM2 protein unlike Nutilis, triggering diverse cell death pathways (Zhao et al. 2013).

For instance, NVP-CGM097 which was concomitantly investigated with an endocrine therapy against several models, resulted in the reactivation of wildtype p53 induced suppression of E2 F targets and G2/M checkpoint signaling and overexpression of p53 dependent apoptotic pathways. These observed data occurred due to simultaneous inhibition of both MDM2 and the CDK4/6 pathway. CDK4/6 inhibition acts to prevent G1 cell cycle entry by preventing the phosphorylation of Rb (Petrossian et al. 2018). The major outcome of the synergism between NVP-CGM097 and fulvestrant was enhanced E2 F downregulation, which resulted in cell cycle arrest (Portman et al. 2020).

In cellular proliferation, ER and p53 have contradictory effects. As a transcriptional regulator, p53 has the power to stimulate or suppress a wide range of target genes. On the other hand, ER impedes p53-mediated cell cycle arrest and apoptosis (Chimento et al. 2022).

Interestingly, the cell death caused by antiestrogens is supposed to have several possible mechanisms (Huang et al. 2020). Overexpression of survivin leads to inhibition of apoptosis. An important mechanism explaining the antiapoptotic function of ERα is transcriptional suppression of survivin. Therefore, wild-type p53 may be functionally repressed by ERα via up-regulation of survivin. ERα may recruit other repressors of the p53 function. In addition, direct binding of ERα to p53 both in vitro and in vivo on endogenous p53 target gene promoters hinders transcriptional activation by p53 (Martínez-Sifuentes et al. 2022). That may explain the weak expression of p53 obtained in Ehrlich control group.

It was demonstrated that ERα controls proliferation of breast cancer cells in MCF- 7 as knockdown of ERα significantly increases p53/p21 expression. This effect is mediated by an estrogen response element (ERE)-dependent manner through formation of the ERα/ERE complex both in vitro and in vivo (Liao et al. 2014). Confirming these observations, a study conducted on EAC revealed that treatment with LQFM030 halted the cells at the G1 phase, activated caspase 7, and increased p53 (da Mota et al. 2016).

In the present study, treatment with alizarin augmented p53 expression accompanied by reduction in MDM2 level suggesting that alizarin could act as an inhibitor to the p53–MDM2 interaction, reactivating p53 and its main roles, cell cycle arrest and apoptosis through its antiestrogenic effect. Consistent with these findings, SZ- 685 C, a murine anthraquinone, structurally similar to alizarin, induced apoptosis leading to antitumor effects in breast cancer in vitro (MCF- 7) and in vivo (Xie et al. 2010), warranting that SZ- 685 C sensitizes breast cancer cells to antiestrogen drugs (Zhu et al. 2012). Another research studying the effect of alizarin on pancreatic cancer reported G2/M phase arrest where the proapoptotic protein cleaved caspase 3 was upregulated in contrast to the antiapoptotic-related proteins p-p65, XIAP, Bcl- 2, and c-myc (Xu et al. 2022).

Supporting the outcomes of the current study, it was shown that NVP-CGM097 induced apoptosis increasing p53 acetylation/activation and cell cycle arrest in G1 and G2, in wild-type p53 ER-positive breast cancer cell line (MCF- 7 and ZR75 - 1) models (Portman et al. 2020). Moreover, MDM2 inhibition was characterized by marked expression of the vital cell cycle regulator p21, which is a target for p53 and chief constituent of cell cycle arrest in G1 and G2 (Fischer et al. 2016).

In the current study, treatment with alizarin raised the expression of Bax and caspase 3. Similarly, in a study made on MCF- 7 cells mesenchymal stem cells significantly inhibited proliferation, stimulated apoptosis via inducing the gene expression of apoptotic p53, upregulating caspase 3/9, pro-apoptotic Bax gene and downregulated the antiapoptotic Bcl- 2 gene (Chao et al. 2012, Felthaus et al. 2024, Mosadegh et al. 2025). It was reported that N9 (novel synthetic chalcone**)** also triggered the production of the pro-apoptotic markers Bax and p53 via suppression of MDM2 (Loch-Neckel et al. 2015).

Another study investigating the role of three anthraquinone derivatives, structurally similar to alizarin came in line with the findings of the present study. The first one activation of apoptosis was correlated to the cleavage of caspase 9 and caspase 3. The second one induced a rise in the Bax level and a fall in the Bcl- 2 level, which in turn activated the mitochondrial pathway, leading to release of cytochrome c, eventually the activation of caspase 9 and caspase 3. The third one enhanced the ROS generation triggering caspase 3 and p53 expression (Yang et al. 2019).

Several lines of evidence reported that the activation of p53 by DNA destructive agents or any other way can reduce the resistance of cells to apoptotic stimuli through p53-mediated effects on bcl2 and bax gene expression. These p53-induced changes would modify the ratio of Bcl- 2 and Bax proteins, employing the cells into a state of increased susceptibility to apoptosis (Sharawi 2020).

These results of the current study were further verified in a study on an alizarin derivative revealing a significant rise in the levels of Bax, caspase 9, and caspase 3 decreasing Bcl- 2 expression. (Huang et al. 2017a, b). Another study investigating the effect of strawberry extracts as an anticancer agent against T47D cells demonstrated a dose dependent increase in p53 in conjunction with caspase 3 and Bax. That was attributed to upregulation of p73 which is an isoform activates p53 responsive genes and regulate cell survival or cell death. Besides, a rise in Bax concentration results in penetration of mitochondrial outer membrane, release of cytochrome-C, thereby activating caspase 9 (Somasagara et al. 2012). Furthermore, some alizarin derivatives activated apoptosis and G1 phase arrest by increasing the production of intracellular Ca + and ROS indicating that these compounds may induce apoptosis through a mitochondrion-dependent pathway (Yao et al. 2014).

## Conclusion

Data obtained from the present study imply that alizarin may represent a new therapeutic modality to combat breast cancer. This activity could be achieved through different mechanisms. Alizarin demonstrated a significant binding affinity to the estrogen receptor (ER), effectively modulating the downstream MDM2/Rb/E2F1 signaling cascade. This observation aligns with the findings from both the MTT assay and in vivo experiments as well as the docking studies. Furthermore, alizarin activated p53, leading to the subsequent induction of apoptosis. Collectively, these findings suggest that alizarin may possess potential antiestrogenic properties.

**Figure:**
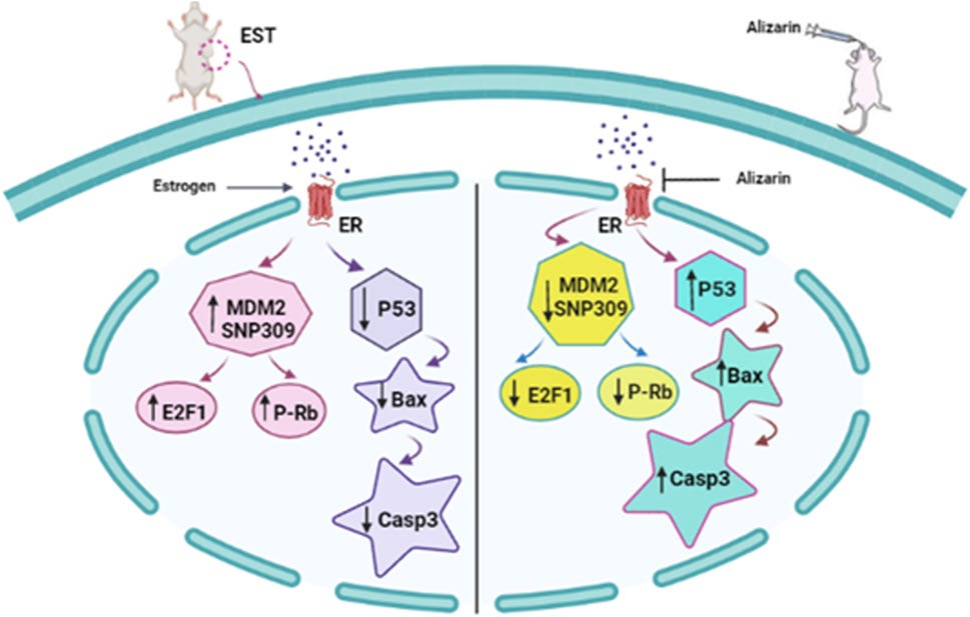
Schematic representation illustrates the micro-environment in the EST with overexpression of MDM2 and activation of E2F1 and hyperphosphorylation of RB because of estrogen receptor signaling; at the same time, the apoptotic function of p53 and its target genes is prohibited due to the overexpression of MDM2. The right side of the graphical abstract represents the mechanistic pathway by which alizarin worked on antagonizing ERα and consequently, MDM2/E2F1/p-Rb axis was downregulated, synchronously; the p53 tumor suppressor gene is liberated and reactivated once again to alleviate its target genes Bax and caspase 3 restoring the apoptotic function

## Data Availability

All source data for this work (or generated in this study) are available upon reasonable request.
